# Dengue epidemic in Burkina Faso: concerns about the informal use of traditional herbal remedies

**DOI:** 10.11604/pamj.2024.47.71.42323

**Published:** 2024-02-19

**Authors:** Kampadilemba Ouoba, Daniel Dori, Rasmané Semdé

**Affiliations:** 1 Laboratory of Drug Development, Centre for Training, Research and Expertise in Drug Sciences, Joseph Ki-Zerbo University, Ouagadougou, Burkina Faso,; 2 National Pharmaceutical Regulatory Agency, Ministry of Health and Public Hygiene, Ouagadougou, Burkina Faso,; 3 Public Health Laboratory, Science and Health Doctoral School, Joseph Ki-Zerbo University, Ouagadougou, Burkina Faso

**Keywords:** Dengue epidemic, traditional herbal remedies, public health issues, Burkina Faso

## To the editors of the Pan African Medical Journal

In the year 2023, dengue fever, a neglected vector-borne tropical disease, has resurfaced in several endemic areas around the world, particularly in Burkina Faso, which is already experiencing a difficult security situation [1]. The figures for this new outbreak are far higher than those seen in the country in 2016 and 2017, with a worrying rise in deaths [2].

According to the weekly epidemiological surveillance report from the Ministry of Health and Public Hygiene, the cumulative number of cases of dengue fever recorded in Burkina Faso from the start of 2023 to 12^th^ November was 109,908, including 511 deaths, giving a case-fatality rate of 0.5%. This far exceeds the cumulative number of deaths linked to COVID-19 in Burkina Faso since the start of the pandemic - around 22,000 cases and 396 deaths [3].

In response to this latest outbreak of dengue fever, the Ministry of Health and Public Hygiene has undertaken a number of vector control measures aimed at stopping the transmission of this arbovirosis. It has also urged the public to refrain from self-medicating with any suspected symptoms of dengue fever, and in particular to avoid using non-steroidal anti-inflammatory drugs such as ibuprofen, diclofenac, aspirin, etc. These drugs carry a high risk of infection. These drugs carry a risk of bleeding, which aggravates the clinical picture in patients with dengue fever. In addition to this risk, which is specific to the context of the dengue epidemic, it is important to emphasize that self-medication is a major public health threat in Burkina Faso [1], fostered by the weakness of the healthcare system, particularly in terms of pharmaceutical regulation.

Despite the efforts of the Ministry of Health, many people in Burkina Faso have informally turned to traditional medicine, particularly medicinal plants and traditional herbal remedies, to treat or prevent dengue fever. Since the start of the epidemic, there has also been a proliferation of advertising messages on social media for traditional plant-based recipes purporting to be effective against dengue fever, in violation of the national regulations in force, particularly those relating to advertising in Burkina Faso. Faced with this worrying situation, the secretary general of the Ministry of Health and Public Hygiene sounded the alarm in a press release issued on November 16, 2023. The press release draws the population's attention to the potential dangers of these so-called traditional remedies, and informs the public that *"... without calling into question the benefits of traditional medicine when properly used, unlicensed traditional pharmacopoeia products have no official recognition attesting to their efficacy, safety and harmlessness"*. It also reminds the public that *"... the practice of traditional medicine is subject to specific regulations in Burkina Faso, which require the players involved to be licensed and integrated into the health system"*.

The population's eagerness to use traditional herbal medicine in the context of the dengue epidemic in Burkina Faso, as was the case with the COVID-19 pandemic, is thought to be linked to the absence of conventional curative treatments for dengue worldwide, the lack of access to biomedical healthcare in the country and the perceived harmlessness of so-called natural remedies. This situation gives rise to enormous public health concerns, apart from those linked to the epidemic itself, because the popular use of traditional remedies is not without health risks, contrary to popular belief. Indeed, it has been reported in the scientific literature that the use of traditional herbal medicines also carries risks of serious adverse effects, hepatotoxicity or renal toxicity (due to the possible presence of potentially nephrotoxic contaminants such as mercury, lead, and arsenic in traditional herbal products) and harmful pharmacokinetic or pharmacodynamic interactions with synthetic medicines when taken concomitantly [4].

In 2020, in the midst of the COVID-19 pandemic, a study based on the general population carried out in four health regions of Burkina Faso revealed the existence of risks associated with the informal use of traditional medicines [5]. According to the study, 85% of the general population of Burkina Faso used traditional medicines for healthcare. This use was statistically associated with the health region; the populations of the country's two largest cities, Ouagadougou and Bobo Dioulasso, which are currently highly endemic for dengue fever, made greater use of traditional medicines than the populations of the other regions. Nearly fifteen percent (15%) of users had experienced adverse events of varying degrees of severity as a result of using traditional medicines. Unfortunately, none of these cases of adverse events were reported to the national pharmacovigilance system. Other risks were identified by this study, in particular the concomitant use of medicinal plants or traditional plant-based medicines with conventional medicines, estimated at 60% of users, involving potential risks of therapeutic failure or the occurrence of serious adverse reactions. Similarly, the use of traditional remedies during pregnancy was observed in 10% of the female user population, which would entail a risk to the health of the mother or child, especially when their safety has not been studied. The same study showed that traditional health practitioners are the main suppliers of traditional medicines used by the population. However, another study carried out in Burkina Faso during the same period highlighted the fact that the phyto-therapeutic practices of traditional health practitioners do not meet the regulatory requirements and good practices applicable to guarantee the quality and safety of the traditional medicines they market, raising concerns about the various health risks that the use of these traditional products may entail [6].

But these risks could be more frequent and more serious in the general population across the country, due to the unregulated use of traditional herbal medicines, given the absence of standardized doses or dosages and the lack of evidence-based data on the efficacy and safety of the plants or plant combinations that make up traditional herbal medicine products.

The informal use of medicinal plants and traditional plant-based medicines in Burkina Faso poses a number of challenges. These include the failure to integrate unregistered traditional medicines into the national health product vigilance system, the failure to formally involve patients or consumers and traditional health practitioners in the national system for reporting adverse drug reactions, and the absence of a formal national program for training and educating traditional health practitioners in the national regulatory requirements for traditional medicine, good practice in the manufacture of herbal medicines and the monitoring of any risks associated with the traditional medicines they market. In addition, there is a lack of an ongoing training and awareness program for conventional healthcare professionals on traditional and complementary medicine, and on monitoring the risks associated with traditional medicine products in their dealings with patients.

## Conclusion

In light of the above, it is clear that the informal use of traditional herbal medicines is a major public health issue in Burkina Faso. Consequently, it is imperative to develop phytovigilance, which is the monitoring of risks associated with the use of medicinal plants and traditional plant-based medicines, as part of the national health product vigilance system. To achieve this, the country must, from a public health perspective, formally integrate traditional medicines, including those that are not registered in the national health product surveillance system. In addition, consumers, patients, and traditional health practitioners must be legal actors in the reporting of adverse drug reactions in this national surveillance system. Also, and especially in the context of epidemics in Burkina Faso, effective and appropriate mechanisms for communicating about the risks associated with the popular use of traditional herbal remedies must be implemented, and this communication must take place in the country's main local languages, in order to achieve tangible results. Health professionals should also be trained and made aware of the need to monitor the use of traditional remedies among patients, particularly those suffering from chronic pathologies. Finally, a sustainable training programme for traditional health practitioners on good manufacturing practices of herbal medicines must be set up to guarantee the quality and safety of traditional herbal medicine products.
